# Amelioration of nitrate uptake under salt stress by ectomycorrhiza with and without a Hartig net

**DOI:** 10.1111/nph.15740

**Published:** 2019-03-14

**Authors:** Gang Sa, Jun Yao, Chen Deng, Jian Liu, Yinan Zhang, Zhimei Zhu, Yuhong Zhang, Xujun Ma, Rui Zhao, Shanzhi Lin, Cunfu Lu, Andrea Polle, Shaoliang Chen

**Affiliations:** ^1^ Beijing Advanced Innovation Center for Tree Breeding by Molecular Design College of Biological Sciences and Technology Beijing Forestry University Box 162 Beijing 100083 China; ^2^ Gansu Provincial Key Laboratory of Aridland Crop Sciences Gansu Agricultural University Lanzhou 730070 China; ^3^ Urat Desert‐Grassland Research Station Northwest Institute of Eco‐Environment and Resources Chinese Academy of Science Lanzhou 730000 China; ^4^ Forest Botany and Tree Physiology University of Goettingen Göttingen 37077 Germany

**Keywords:** MAJ, NaCl, NAU, NO_3_^−^ flux, NRTs, *Paxillus involutus*, pH, Poplar

## Abstract

- Salt stress is an important environmental cue impeding poplar nitrogen nutrition. Here, we characterized the impact of salinity on proton‐driven nitrate fluxes in ectomycorrhizal roots and the importance of a Hartig net for nitrate uptake.
- We employed two *Paxillus involutus* strains for root colonization: MAJ, which forms typical ectomycorrhizal structures (mantle and Hartig net), and NAU, colonizing roots with a thin, loose hyphal sheath. Fungus‐colonized and noncolonized *Populus* × *canescens* were exposed to sodium chloride and used to measure root surface pH, nitrate (NO_3_^−^) flux and transcription of NO_3_^−^ transporters (NRTs; *PcNRT1.1*, ‐*1.2*, ‐*2.1*), and plasmalemma proton ATPases (HAs; *PcHA4*, ‐*8*,* ‐11*).
- *Paxillus* colonization enhanced root NO_3_^−^ uptake, decreased surface pH, and stimulated *NRT*s and *HA4* of the host regardless the presence or absence of a Hartig net. Under salt stress, noncolonized roots exhibited strong net NO_3_^−^ efflux, whereas beneficial effects of fungal colonization on surface pH and HAs prevented NO_3_^−^ loss. Inhibition of HAs abolished NO_3_^−^ influx under all conditions.
- We found that stimulation of HAs was crucial for the beneficial influence of ectomycorrhiza on NO_3_^−^ uptake, whereas the presence of a Hartig net was not required for improved NO_3_^−^ translocation. Mycorrhizas may contribute to host adaptation to salt‐affected environments by keeping up NO_3_^−^ nutrition.

## Introduction

Soil salinization is a global agricultural and ecological problem (Hasegawa, 2013). Soil degradation by salt is becoming more serious because of inappropriate irrigation and fertilization practices, leading to secondary soil salinization and eventually the loss of arable land (Pitman & Läuchli, 2002). In addition to limiting water uptake and ion‐specific toxicity, high sodium chloride (NaCl) results in competitive interactions with essential mineral nutrients, decreasing nutrient availability, impairing nutrient uptake, and leading to nutritional imbalances in trees (Polle & Chen, 2015).

Nitrogen (N) plays an important role in the amelioration of salt tolerance (Gómez *et al*., 1996; Mansour, 2000). N fertilization alleviates salt stress impacts by compensating and correcting nutritional imbalances in higher plants (Gómez *et al*., 1996). N‐fed poplar trees display a pronounced enrichment of N‐containing compounds, mainly amino acids, upon salt exposure (Dluzniewska *et al*., 2007; Ehlting *et al*., 2007). Accumulation of nitrogenous compounds has been suggested to contribute to osmoprotective processes, safeguarding folded macromolecular protein structures, and mitigating oxidative stress by scavenging reactive oxygen (O) species (Mansour, 2000). Enhanced nitrate (NO_3_^−^) nutrition assists poplar trees (Dluzniewska *et al*., 2007; Ehlting *et al*., 2007) and herbaceous species, such as *Pisum sativum*,* Helianthus annuus*, and *Glycine max* (Bourgeais‐Chaillou *et al*., 1992; Ashraf & Sultana, 2000; Frechilla *et al*., 2001), adapting to saline conditions. Furthermore, processes related to increased NO_3_^−^ uptake and assimilation are stimulated under salt stress; for example, *Salicornia europaea* promotes NO_3_^−^ uptake rate and activity of the nitrate reductase (NR) upon NaCl exposure (Nie *et al*., 2015). Gene expression analyses indicated that salt can stimulate the expression of NO_3_^−^ transporters (NRTs; e.g. *McNRT1* in *Mesembryanthemum crystallinum*; Popova *et al*., 2003). NR activity increases in leaves of hydroponically grown tomato plants in response to salt stress (Cramer & Lips, 1995). Therefore, it has been suggested that procedures aiming at improved plant N nutrition, such as ectomycorrhization, may hold great promise for enhancing salinity tolerance (Chen *et al*., 2014).

In the symbiosis with ectomycorrhizal (EM) fungi, host plants benefit from improved N nutrition (Read, 1991; Nehls & Plassard, 2018). The extramatrical mycelium extending from the EM root tips generally enhances the capacity for uptake of inorganic N compared with nonmycorrhizal (NM) root tips (Melin & Nilsson, 1952, 1953; Finlay *et al*., 1988, 1989; Arnebrant *et al*., 1993). Flux analyses, however, showed divergent effects on NO_3_^−^ uptake for different EM–tree species associations – increase: *Rhizopogon roseolus–Pinus pinaster* (Plassard *et al*., 2002; Gobert & Plassard, 2002); decrease: *Hebeloma cylindrosporum–P. pinaster* (Plassard *et al*., 2002), *Paxillus involutus–Picea abies* (Boukcim & Plassard, 2003). Thus, it is unclear how EM fungi influence NO_3_^−^ uptake and assimilation in salt‐stressed host plants.

Here, our goals were to study the impact of EM on NO_3_^−^ uptake under salt stress and to find out whether the Hartig net, the exchange structure for nutrients between fungus and plant, is required for an effective improvement of NO_3_^−^ flux and plant NO_3_^−^ assimilation. For this purpose, we exploited two different strains of the EM fungus *P. involutus*: MAJ and NAU. Strain MAJ exhibits typical EM characteristics with hyphal mantle and Hartig net (Gafur *et al*., 2004) and can improve whole‐poplar N content and salt tolerance (Langenfeld‐Heyser *et al*., 2007; Luo *et al*., 2009). *Paxillus involutus* strain NAU, which is genetically closely related to *P. involutus* strain MAJ (Le Quéré *et al*., 2004), cannot penetrate the host cell walls and forms only a loose hyphal web around root tips (poplar: Gafur *et al*., 2004; birch: Le Quéré *et al*., 2004). Both strains have positive effects on plant vitality (Müller *et al*., 2013). Here, we combined NO_3_^−^ flux analyses in noncolonized, MAJ‐, or NAU‐colonized root tips with expression analyses of NRTs and determination of NR and nitrite reductase (NiR) activities in poplar (*Populus *×* canescens*). *P. involutus* increases the proton (H^+^)‐pumping activity in mycorrhizal roots (Li *et al*., 2012a; Ma *et al*., 2014a; Zhang *et al*., 2017). The H^+^‐motive force generated by plasmalemma H^+^‐ATPases (PM HAs) contributes to the activation of the NO_3_^−^/H^+^ symport system (Santi *et al*., 1995; Miller & Smith, 1996; Santi, 2003; Britto & Kronzucker, 2006; Sorgonà *et al*., 2010, 2011; Yu *et al*., 2016). Therefore, the contribution of PM HAs to modify NO_3_^−^ fluxes was also assessed in NAU‐ and MAJ‐colonized roots. Our data show that inoculation with *P. involutus* stimulates net NO_3_^−^ influx and that the presence of a Hartig net is not required for this effect. Under salt stress, fungus‐colonized roots retain NO_3_^−^ uptake regardless of the presence of a Hartig net, whereas noncolonized roots show net NO_3_^−^ efflux.

## Materials and Methods

### Fungal culture and salt exposure

The *P. involutus* strains MAJ and NAU from the stock collection of the department of Forest Botany and Tree Physiology (University of Goettingen, Germany) were used for this study. The strains MAJ and NAU were cultured on Petri dishes containing modified Melin Norkrans (MMN) agar medium with the following components: 3.68 mM potassium dihydrogen phosphate, 1.89 mM ammonium sulfate, 0.61 mM magnesium sulfate (MgSO_4_), 0.34 mM calcium chloride (CaCl_2_), 0.43 mM NaCl, 0.06 mM iron(III) chloride, 0.0003 mM thiamine hydrochloride, 55.5 mM glucose, and additional 3 g l^−1^ malt extract (pH 5.2, Gafur *et al*., 2004). The Petri dishes (diameter, 90 mm) were sealed with a strip of Parafilm (Bemis Company Inc., Neenah, WI, USA) and kept in darkness at 23°C. After 1 month of incubation, agar blocks of 5–10 mm length and width containing hyphae were cut with a razor blade, then subjected to 0 or 100 mM NaCl in modified MS nutrient solution with macro elements (12.36 mM potassium nitrate (KNO_3_), 0.81 mM MgSO_4_, 1.30 mM ammonium dihydrogen phosphate, 0.68 mM CaCl_2_), trace elements (5.0 μM potassium iodide, 100 μM boric acid, 100 μM manganese sulfate , 30 μM zinc sulfate , 1.0 μM sodium molybdate, 0.1 μM copper sulfate, 0.1 μM cobalt(II) chloride, 100 μM EDTA disodium salt, 100 μM iron(II) sulfate), and organic substances (0.56 μM inositol, 27 μM glycine, 0. 33 μM thiamine hydrochloride, 2.4 μM pyridoxine hydrochloride, 4.1 μM nicotinic acid), pH 5.3. Steady‐state fluxes of NO_3_^−^ were recorded in these MAJ and NAU hyphae after a short‐term (ST, 24 h) or long‐term (LT, 7 d) salt exposure. The modified MS nutrient solution was also applied to poplars, but with a concentration of 0.1 mM KNO_3_ during the period of hydroponical acclimation before ^15^KNO_3_ labelling and salt treatment (see the ^15^N‐nitrate‐labelling experimental setup and isotope analysis section).

### *Paxillus involutus* colonization of *P. *×* canescens*

Regenerated plantlets of *P. *×* canescens* (syn. gray poplar, a hybrid of *Populus tremula *×* P. alba*) were grown for 3–4 wk on the modified MS medium (see previous section) but with half‐strength macronutrients for rooting (Leplé *et al*., 1992). Uniform plantlets with sufficient roots were used for subsequent fungal inoculation as described by Müller *et al*. (2013). A Petri dish culture system was used for the colonization of *P. *×* canescens* with *P. involutus* strains MAJ or NAU (Müller *et al*., 2013). Rooted plantlets were placed on the MMN agar medium in the presence or absence of fungal mycelium. During the establishment of the symbiosis, the Petri dishes were sealed with Parafilm and covered with aluminum foil to keep the roots in darkness. The temperature in the climate chamber was maintained at 23 ± 1°C with a light period of 16 h (06:00–22:00 h). Photosynthetic active radiation (PAR) of 200 μmol m^−2^ s^−1^ was supplied by cool white fluorescent lamps. After 30 d of inoculation, fungus‐colonized and NM plants with similar height and growth performance were used for NaCl treatment. For anatomical analysis, MAJ‐ and NAU‐colonized roots were embedded and sectioned with a dry glass knife on an ultramicrotome (Ultracut E; Reichert‐Jung, Vienna, Austria). Semi‐thin cross‐sections of 500 nm were stained with 0.1% (w/v) toluidine blue in 0.1% (w/v) disodium tetraborate decahydrate and photographed as described previously (Li *et al*., 2012a).

### ^15^N‐nitrate‐labelling experimental setup and isotope analysis

NM and mycorrhizal plants were placed in modified MS nutrient solution containing 0.1 mM KNO_3_ for 2 d of acclimation. Then, poplars were transferred to modified MS nutrient solution with 1 mM K^15^NO_3_ (^15^N enrichment 99%; Cambridge Isotope Laboratories Inc., Andover, MA, USA). Nonlabelled controls were supplied with 1 mM KNO_3_ in the nutrient solution. After 24 h exposure the plants were removed, the roots were washed, and leaves, stems, and roots were dried at 40°C for 120 h.

Nonlabelled and labelled samples were ground in a ball mill (GT200; Beijing Grinder Instrument Co. Ltd, Beijing, China). For stable isotope analysis, 6–8 mg DW of roots, stem, and leaves were filled into tin cartouches and analyzed with a coupled system consisting of a Vario Pyro tube element analyzer (Elementar Analysensysteme GmbH, Langenselbold, Germany) and an IsoPrime‐100 stable isotopic ratio mass spectrometer (Elementar UK, Stockport, UK). Abundances of ^15^N were expressed using the *δ* notation with *δ*_sample_ (‰) = [(*R*_sample_ − *R*_standard_)/*R*_standard_] × 1000; *R*_sample_ and *R*_standard_ represent the ^15^N : ^14^N ratio of samples and standard, respectively. Atmospheric ^15^N abundance served as the primary standard. Data were used to calculate the enrichment of ^15^N (atomic percent) in nonlabelled and labelled samples. The enrichment of ^15^N (APE, atomic percent excess) was calculated as

$${}^{15}\text{N APE}={}^{15}N{\left(\text{at.}\%\right)}_{\mathrm{labelled}}{-}^{15}N{\left(\text{at.}\%\right)}_{\mathrm{nonlabelled}}$$

### Salt treatment

Fungus‐colonized and NM plants were carefully removed from MMN agar medium for hydroponic culture. The plants were transferred into modified MS nutrient solution containing 0.1 mM KNO_3_ with continuous aeration. Temperature was maintained at 23°C, with a light period of 16 h (06:00–22:00 h), and PAR was 200 μmol m^−2^ s^−1^. After 2–3 d of acclimation, these plants were exposed to 0 or 100 mM NaCl in MS solution either for an ST (24 h) or an LT (7 d) treatment. The solutions were regularly renewed (every 24 h). After 24 h or 7 d of salt treatment, root surface pH, fluxes of NO_3_^−^, activities of NR and NiR, and transcript levels of genes encoding NRTs and PM HAs were examined.

Fungal mycelia were pretreated in the same media as the plants. The youngest and active hyphae were used for salt treatment and subsequent flux and pH recordings. During the period of salt treatment, agar plugs with emanating hyphae were incubated in modified MS solution in the presence or absence of 100 mM NaCl for ST or LT treatment. Before flux recordings, agar plugs were transferred into 150 ml of measuring solutions and incubated on a rotary shaker (150 rpm, 23°C) for 1 h in darkness. Thereby, the agar plug was equilibrated with the measuring solution. The pH and flux of NO_3_^−^ were recorded in control and NaCl‐treated fungal mycelia.

### Root surface pH

The root surface pH was determined using H^+^ selective microelectrodes (as described in the following section). pH values were calculated from an established relationship between pH values and electrical potentials at the root surface (*r* ^2^ > 0.999) as previously described (Sun *et al*., 2009a).

### Net flux of NO_3_^−^ and H^+^ and pH measurements 

pH values and flux profiles of NO_3_^−^ and H^+^ were measured with the noninvasive micro‐test technique (NMT‐YG‐100; Younger USA LLC, Amherst, MA, USA) with aset 2.0 (Sciencewares, Falmouth, MA, USA) and ifluxes 1.0 software (Younger USA). The silanization of glass micropipettes and preparation of microelectrodes were previously described (Sun *et al*., 2009a,b). The micropipettes were front‐filled with 15 μm columns of selective liquid ion‐exchange cocktails (H: Fluka 95293; Fluka Chemie GmbH, Buchs, Switzerland; NO_3_^−^: XY‐SJ‐NO_3_^−^; Younger USA). Ion‐selective microelectrodes were calibrated by the following target ion's standard solution before flux recordings:

1. H^+^ microelectrodes – pH 4.5, 5.5 and 6.5 (pH of the measurement solution was adjusted to 5.3);
2. NO_3_^−^ microelectrodes – 0.05, 0.1 and 0.5 mM NO_3_^−^ (NO_3_^−^ was 0.1 mM in the measurement solution).

Only electrodes with Nernstian slopes > 58 ± 5 mV decade^−1^ were used in our noninvasive micro‐test. pH values on the root surface were calculated on the basis of the electrical potentials at the root surface. The ion flux rates of NO_3_^−^ and H^+^ were calculated on the basis of Fick's law of diffusion (Sun *et al*., 2009a,b).

### Steady‐state ion flux and pH measurements

Roots segments with the apices of 15–20 mm lengths were sampled from controls, ST‐, and LT‐salt stressed plants of *P. *×* canescens* colonized with or without *P. involutus*. The roots were rinsed with distilled water, equilibrated for 40 min in the basic measuring solution containing 0.1 mM KNO_3_, and then transferred to the measuring chamber containing 5 ml of fresh measurement solution and immobilized at the bottom of the measuring chamber. Fluxes of NO_3_^−^ and H^+^ and pH were measured along roots in the following solutions:

1. NO_3_^−^ measuring solution – 0.1 mM KNO_3_, 0.1 mM potassium chloride (KCl), 0.1 mM CaCl_2_, pH 5.3 was adjusted with potassium hydroxide (KOH) and hydrochloric acid (HCl);
2. H^+^ measuring solution – 0.1 mM NaCl, 0.1 mM MgCl_2_, 0.1 mM CaCl_2_ and 0.5 mM KCl, pH 5.3 was adjusted with KOH and HCl.

The pH and steady‐state flux recordings of NO_3_^−^ and H^+^ started at a distance of 100 μm from the apex and were conducted along the root axis until 2100 μm from the apex at intervals of 100–300 μm. For pH measurements, electrical potentials along the roots were recorded with H^+^ microelectrodes at the same distances. For flux recording, the concentration gradients of NO_3_^−^ and H^+^ ions were measured by moving the ion‐selective microelectrode between two positions (30 μm in distance) close to the root surface at a programmable frequency in the range of 0.3–0.5 Hz. At each measuring point the recording was continued for 6–8 min (Li *et al*., 2012a; Lu *et al*., 2013; Sun *et al*., 2013a,b). The measuring interval was long enough to cover oscillatory periods of the measured ions (Shabala *et al*., 2006; Zhang *et al*., 2017).

### Stability of H^+^ microelectrodes to root manipulation

To testify whether the H^+^ microelectrodes are stable to root manipulation, the variations of pH and H^+^ flux were recorded as a function of time at 300 or 400 μm from the root tip because pH values and H^+^ flux at these positions are the most variable in NM or mycorrhizal roots. The recordings of the microelectrodes demonstrated that the signals were stable during the 60 min measuring period on roots colonized with or without *Paxillus* strains (Supporting Information Fig. S1).

### Sensitivity of H^+^ microelectrodes in NO_3_^−^ or in H^+^ measuring solutions

The sensitivity of H^+^ microelectrodes to pH changes was examined by calibrating the electrodes at pH 4.5, 5.5 and 6.5 in NO_3_^−^ or in H^+^ measuring solutions. The calibration characteristics (Nernst slope and intercept) of the H^+^ electrodes did not differ between NO_3_^−^ and H^+^ measuring solutions (Table S1).

### Inhibition of PM HAs

Fungus‐colonized and NM roots collected from control and NaCl‐stressed poplars were pretreated without or with sodium orthovanadate (PM HA inhibitor, 500 μM; Sun *et al*., 2010). After 40 min of inoculation, root surface pH and steady‐state fluxes of NO_3_^−^ and H^+^ were examined along the root tips from 100 to 2100 μm from the apex.

### Membrane potential and O flux measurement

NM and mycorrhizal plants were hydroponically acclimated for 2 d in modified MS nutrient solution containing 0.1 mM KNO_3_, and then placed in modified MS nutrient solution for 24 h. The root tips were rinsed with distilled water and transferred to H^+^ measuring solution for 40 min equilibration. Membrane potential at the apical region (300 or 400 μm from the tip) was detected with silver/silver chloride microelectrodes (XY‐CGQ‐03; Xuyue (Beijing) Sci. & Tech Co. Ltd, Beijing, China) (Li *et al*., 2012b). O fluxes along roots were measured based on the noninvasive micro‐test technique with O microelectrodes (XY‐CGQ‐501; Younger USA) (Li *et al*., 2014).

### Determination of NR and NiR activities

NR activity was determined with a Micro NR Assay Kit (NR‐BC0085) and NiR activity with an NiR Assay Kit (BC1545; Beijing Solarbio Science & Technology Co. Ltd, Beijing, China) following the manufacturer's instructions. In brief, fungus‐colonized and NM roots (0.1 g FW) were sampled from control and salt‐exposed plants (100 mM NaCl for 24 h or 7 d), homogenized in a cold mortar with a pestle in 1 ml extraction solution. The homogenate was centrifuged at 4000 ***g*** (NR) or 10 000 ***g*** (NiR) for 10 min at 4°C. Immediately after centrifugation, the supernatant was added to reaction mixtures designed for NR (EC 1.7.1.3) or for NiR (EC 1.7.1.1) activities. After incubation at 25°C for 30 min (NR) or 37°C for 1 h (NiR), the mixtures were measured at 540 nm with a Tecan Infinite M200 Pro Multimode Microplate Reader (AZoNetwork UK Ltd, Manchester, UK).

### Determination of NRT and PM HA by real‐time quantitative PCR

After exposure of fungus‐colonized and NM plants to 100 mM NaCl for 24 h or 7 d, total RNA was isolated from the control and salt‐stressed roots with RN53‐EASYspin Plus (Aidlab Biotechnologies, Beijing, China) according to the manufacturer's instructions. The amount and purity of isolated RNA were determined spectrophotometrically with a Nano Drop 2000 (Thermo Fisher Scientific Inc., Wilmington, DE, USA). An oligo (dT) primer (Promega, Madison, WI, USA) and Moloney Murine Leukemia Virus reverse transcriptase were used for RNA (1 μg) reverse transcription. The complementary DNA products were used for real‐time quantitative PCR (RT‐qPCR) amplification. The gene‐specific primers for genes of NRTs (*PcNRT*s, *PcNRT1.1*, ‐*1.2*, ‐*2.1*) (Bai *et al*., 2013; Xuan *et al*., 2017) and PM HA (*PcHA*s, *PcHA4*, ‐*8*, ‐*11*; Vitart *et al*., 2001; Palmgren, 2001; Sherrier *et al*., 2005; Rodrigues *et al*., 2014) are shown in Table S2. The *PcUBQ‐L* gene was used as an internal control for *P. *×* canescens*, and RT‐qPCR amplification was performed as described in Shen *et al*. (2015).

### Data analysis

Net ion flux data were calculated with the program Jcal v.3.2.1, a free MS excel‐like spreadsheet, which was developed by the Yue Xu (http://www.xuyue.net/). All experimental data were subjected to ANOVA. Comparisons between means were performed with Duncan's multiple range tests. The program Statgraphics centurion XVI (v.16.1.15; Statgraphics Technologies Inc., The Plains, VA, USA) was used to compare regression lines. Unless stated otherwise, means with *P* < 0.05 were considered to be significantly different.

## Results

### NO_3_^−^ uptake and metabolism

**Figure 1:**
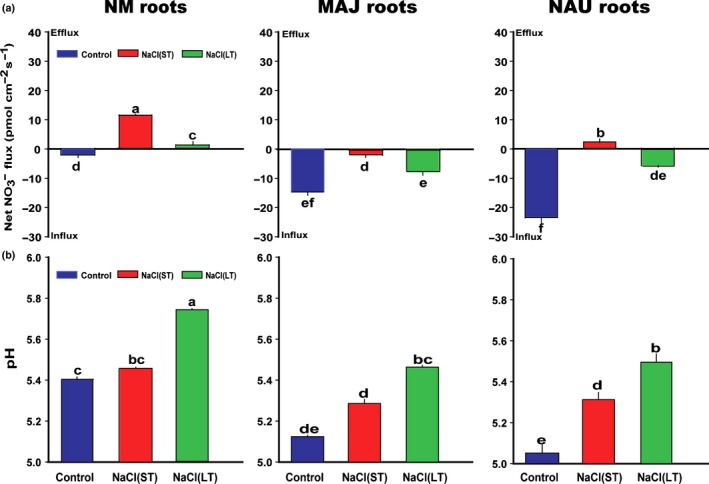
Effects of sodium chloride (NaCl) on steady‐state fluxes of nitrate (NO_3_^−^) and root surface pH in *Populus *×* canescens* colonized without (nonmycorrhizal, NM) or with *Paxillus involutus* strains MAJ and NAU. *P. × canescens* roots were inoculated without or with the *P. involutus* strains, MAJ and NAU, for 30 d, respectively. NM and fungus‐colonized *P. × canescens* plants were exposed to 0 or 100 mM NaCl for 24 h (short term, ST) or 7 d (long term, LT) in MS nutrient solution. (a, b) Mean values of NO_3_^−^ and pH that were measured along root axis, 100–2100 μm from the apex, at intervals of 100–300 μm (Supporting Information Fig. S3). Each column is the mean of five or six individual plants, and bars represent the SEM. Columns labelled with different letters indicate significant differences at *P *<* *0.05 between treatments.

MAJ‐colonized poplar roots showed a thick mantle of fungal hyphae and a well‐developed Hartig net between the first two to three layers of cortex cells, whereas the NAU‐colonized roots showed a thin hyphal mantle and no fungal cells intruding into the root cortex (Fig. S2). Exposure of poplars to ^15^N‐NO_3_^−^ in the nutrient solution resulted in higher enrichment of ^15^N in roots and stems of poplars colonized with *P. involutus* strain MAJ or NAU than in NM poplars (Table 1). ^15^N enrichment did not differ between NAU‐ and MAJ‐colonized plants (Table 1). Since ^15^N tracing does reveal the identity of the transported N compound, we used microelectrodes to determine NO_3_^−^ fluxes in the presence or absence of fungal colonization and in response to salt stress. Along the root apex (100–2100 μm), the fluxes of NO_3_^−^ were constant in the NO_3_^−^ measuring solution (0.1 mM low NO_3_^−^, Fig. S3a). The magnitude and direction of NO_3_^−^ fluxes were significantly influenced by salt exposure and fungal colonization (Figs 1a, S3a). NM root tips showed a moderate NO_3_^−^ uptake, whereas fungus‐colonized roots exhibited 7.4‐ to 11.8‐fold higher uptake (Fig. 1a). Apparently, a Hartig net was not required for this stimulation because root colonization with either *P. involutus* strain MAJ or NAU resulted in enhanced NO_3_^−^ uptake compared with NM roots (Fig. 1a). Furthermore, fungus‐colonized roots maintained net NO_3_^−^ uptake under salinity, whereas NM roots showed net NO_3_^−^ release under ST and LT salt exposure (Fig. 1a). Pure fungal mycelia of NAU and MAJ exhibited net NO_3_^−^ influx irrespective of control or salt treatments (Fig. S4a).

**Table 1:** Nitrogen‐15 (^15^N) enrichment (atomic percent excess) in *Populus *×* canescens* colonized without (nonmycorrhizal, NM) or with *Paxillus involutus* strains MAJ or NAU

	NM	SE		NAU	SE		MAJ	SE
Root	0.202	0.076 a		0.516	0.085 ab		0.639	0.142 b
Stem	0.203	0.055 a		0.567	0.102 b		0.418	0.126 ab
Leaf	0.237	0.097 a		0.243	0.020 a		0.325	0.169 ab

NM and *Paxillus*‐colonized plants were exposed for 24 h to ^15^N‐labelled potassium nitrate (K^15^NO_3_, 1 mM) in modified MS nutrient solution. Controls were exposed to nonlabelled KNO_3_. Tissues were separated and used for stable isotope analyses. ^15^N enrichment was determined as ^15^N (at.%)_labelled_ − ^15^N (at.%)_nonlabelled_. Each value is the mean of three individual plants (± SE). Values labelled with different letters indicate significant differences at *P *<* *0.05.

**Figure 2:**
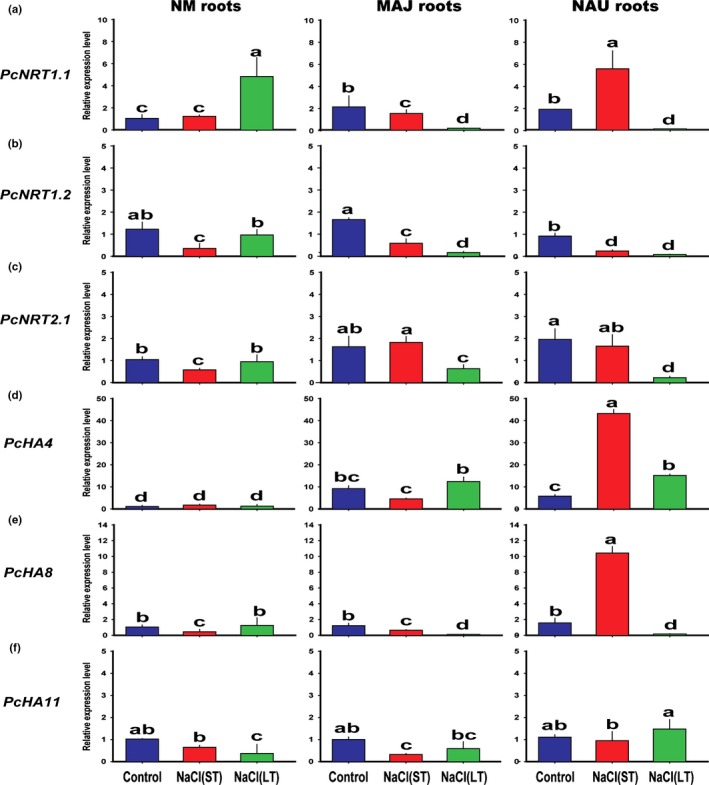
Effects of sodium chloride (NaCl) on expression profiles of nitrate transporters (*PcNRT*s) and plasmalemma H^+^‐ATPases (*PcHA*s) in *Populus *×* canescens* roots colonized without (nonmycorrhizal, NM) or with *Paxillus involutus* strains MAJ and NAU. *P. *× *canescens* roots were inoculated without or with the *P. involutus* strains, MAJ and NAU, for 30 d, respectively. NM and fungus‐colonized *P. *×* canescens* plants were exposed to 0 or 100 mM NaCl for 24 h (short term, ST) or 7 d (long term, LT) in MS nutrient solution. Real‐time quantitative PCR (RT‐qPCR) was performed using *PcUBQ‐L* gene as an internal control. Specific RT‐qPCR primers designed to target *PcNRT1.1*,* PcNRT1.2*,* PcNRT2.1*,* PcHA4*,* PcHA8*,* PcHA11*, and reference genes are shown in Supporting Information Table S2. Each column is the mean of three independent experiments and bars represent the SEM. Columns labelled with different letters indicate significant differences at *P *<* *0.05 between treatments.

Fungal colonization and salt stress also changed the transcript levels of low‐ and high‐affinity NRTs in poplar roots (Fig. 2a–c). Under control conditions, *PcNRT1.1*, whose *Arabidopsis* orthologue also functions as an NO_3_^−^ receptor (Ho *et al*., 2009) and *PcNRT2.1* transcript levels, which encode high‐affinity transporters, were higher in fungus‐colonized than in NM roots (Fig. 2a,c). *PcNRT1.2*, a putative, low‐affinity transporter, showed no clear change in comparison with NM roots (Fig. 2b). ST salt exposure had divergent effects on *PcNRT1.1* transcript levels, with increases in NAU‐ and decreases in MAJ‐colonized roots and no effects in NM roots (Fig. 2a). LT salt exposure caused a strong increase in *PcNRT1.1* in NM roots but decreased the transcript abundances in fungus‐colonized roots (Fig. 2a). Similarly, the *PcNRT1.2* transcript levels were also lower in MAJ‐ and NAU‐colonized roots than in NM roots after LT salt exposure (Fig. 2b). Transcript levels of *PcNRT2.1* decreased in NM roots after ST salt exposure but recovered after LT salt exposure. In roots colonized with MAJ or NAU, *PcNRT2.1* did not respond to ST salt stress but decreased after LT salt exposure (Fig. 2c).

**Figure 3:**
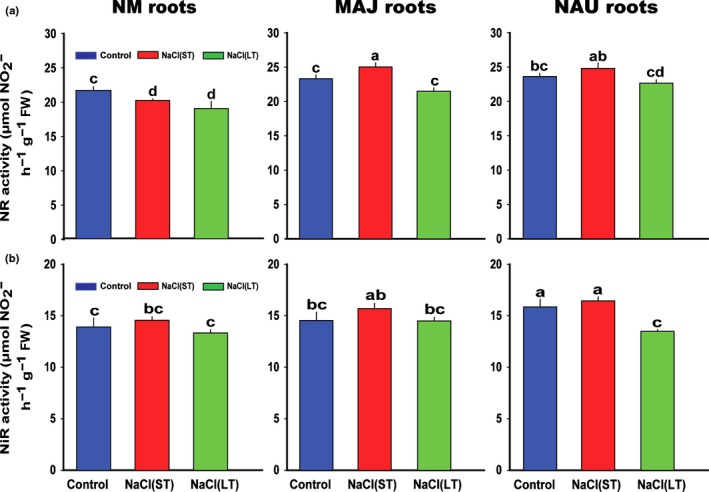
Effects of sodium chloride (NaCl) on activity of nitrate reductase (NR) and nitrite reductase (NiR) in *Populus × canescens* roots colonized without (nonmycorrhizal, NM) or with *Paxillus involutus* strains MAJ and NAU. *P. × canescens* roots were inoculated without or with the *P. involutus* strains, MAJ and NAU, for 30 d, respectively. Root NR and NiR activities were examined after NM and fungus‐colonized *P. × canescens* plants were exposed to 0 or 100 mM NaCl for 24 h (short term, ST) or 7 d (long term, LT) in MS nutrient solution. Each column is the mean of three independent experiments and bars represent the SEM. Columns labelled with different letters indicate significant differences at *P *<* *0.05 between treatments.

The activities of NR and NiR in NM roots were similar to those in fungus‐colonized roots and unaffected by ST salt stress (NR: 23.08 ± 0.35 μmol h^−1^ g^−1^; NiR: 16.17 ± 0.39 μmol h^−1^ g^−1^; Fig. 3). After LT salt exposure, the activities of these enzymes showed a moderate decrease (NR: 21.03 ± 0.46 μmol h^−1^ g^−1^, *P *<* *0.001; NiR: 13.77 ± 0.25 μmol h^−1^ g^−1^, *P *=* *0.065; Fig. 3).

### Root surface pH and HAs as drivers of NO_3_^−^ uptake

NO_3_^−^ uptake requires H^+^ cotransport and is therefore dependent on the pH in the external environment (Rao & Rains, 1976; Doddema & Telkamp, 1979; Fuggi, 1985; Ullrich & Novacky, 1990; Meharg & Blatt, 1995). Here, we determined the pH values at NM and fungus‐colonized root surfaces. The pH along NM roots was stable (Fig. S3b), with a mean value of 5.41 (Fig. 1b). Fungal colonization resulted in a more acidic surface pH, ranging from 5.05 to 5.12 (Figs 1b, S3b) as the result of higher net H^+^ efflux from the surface of fungus‐colonized than from NM roots (Fig. S5; Ramos *et al*., 2009). LT salinity caused a marked rise of pH in NM plants to about pH 5.8 (*P *<* *0.001). In fungus‐colonized plants, salt exposure also caused pH increments resulting in pH values of *c*. 5.4 at the root surface similar to those of NM control roots (Figs 1b, S3b). The salt‐induced increase of pH was due to the decline of H^+^ efflux from root surface (Fig. S5). The surface pH of NAU did not differ from that of MAJ‐colonized roots regardless of control conditions, or ST or LT salt stress (Fig. 1b).

**Figure 4:**
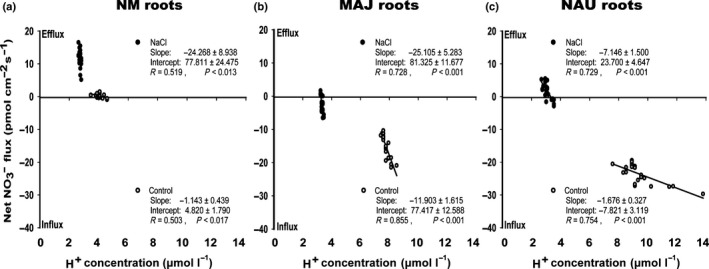
The correlation between nitrate (NO_3_^−^) fluxes and root surface proton (H^+^) concentrations in *Populus *×* canescens* colonized without (nonmycorrhizal, NM) or with *Paxillus involutus* strains MAJ and NAU in the absence and presence of sodium chloride (NaCl). (a) NM roots; (b) MAJ roots; (c) NAU roots. NM and fungus‐colonized *P. *×* canescens* plants were subjected to MS nutrient solution supplemented with 0 or 100 mM NaCl for hydroponic cultures (24 h to 7 d). NO_3_^−^ fluxes and pH were measured along root axis, 100–2100 μm from the apex, at intervals of 100–300 μm. Each point is the mean of five or six individual plants.

The NO_3_^−^ fluxes showed highly significant linear correlations with [H^+^] on the root surface in the absence and presence of NaCl stress (Fig. 4), supporting that NO_3_^−^ uptake of fungus‐colonized roots is dependent on H^+^ gradients as that of NM roots. We noticed that the curves showed differences in the slopes and also parallel curve shifts (translation; Fig. 4). Changes in the slopes of NO_3_^−^ flux/[H^+^] can be interpreted as changes in pH sensitivity of the net flux, which can, for example, be caused by changes in the affinity for NO_3_^−^ or an altered H^+^/NO_3_^−^ stoichiometry. Translation towards the negative direction indicates a change in the ratio of efflux to influx, which can, for example, be achieved by higher numbers of transporters. Under control conditions, the slopes of NM and NAU‐colonized roots were similar (*P* = 0.644), but the NAU curve shifted towards higher net uptake (Fig. 4a,c). The MAJ‐colonized roots showed a steeper slope than NM or NAU‐colonized roots (*P* < 0.001, Fig. 4b) and also a shift towards high net uptake (Fig. 4b). Salt exposure increased the slopes of NM and fungus‐colonized roots (Fig. 4), resulting in the same curves for NM and MAJ‐colonized roots (*P*_intercept_ = 0.756, *P*_slope_ = 0.935). Under salt stress, the curve for NAU‐colonized roots was less steep than that of NM or MAJ‐colonized roots (*P*_NM vs NAU_ = 0.035, *P*_MAJ vs NAU_ = 0.003) but steeper than in the absence of salt (*P*_control vs salt_ = 0.002) (Fig. 4c). Furthermore, under salt stress, the curves of fungus‐colonized roots showed an upward shift, close to the compensation point where influx equals efflux. Since both fungal strains were able to maintain lower surface pH values than NM roots, a small net NO_3_^−^ influx was still found (Fig. 4). This suggests that lower root surface pH in the presence of fungal colonization served as a protection mechanism for plant NO_3_^−^ uptake under salt stress.

Pure fungal mycelium had a lower surface pH than that of NM roots, regardless of the presence or absence of salt (Fig. 1, NM; Fig. S4b, fungal mycelium). Here, we tested whether fungal colonization can also potentially influence the plant's ability for H^+^ extrusion. An acidic environment at the surface of roots is maintained by electrogenic H^+^ extrusion by HAs in the plasma membrane (Palmgren, 2001). Transcript levels of *PcHA*s were examined in NM and fungus‐colonized roots. The analyzed *HA*s include the highly expressed poplar *HA8* (Nilsson *et al*., 2010) as well as *HA4* and *HA11*, whose Arabidopsis/poplar orthologues contribute to stress tolerance (Vitart *et al*., 2001; Gévaudant *et al*., 2007; Dumont *et al*., 2014). Among the three *HA* genes tested, *PcHA4* showed higher transcript levels in fungus‐colonized than in NM roots, whereas *PcHA8* and *PcHA11* transcript abundances did not differ among colonized and NM roots under control conditions (Fig. 2d–f). This observation is in agreement with the lower surface pH values on fungus‐colonized than on NM roots under control conditions (Fig. 1). Salt stress had either no effect on *PcHA* transcript abundances or caused reductions, with the exception of *PcHA4* and *PcHA8* in salt‐stressed roots colonized with NAU (Fig. 2d–f). Under ST salt stress, strong upregulation of *PcHA4* and *PcHA8* was found (Fig. 2d–f), which did not prevent an increase in pH (Fig. 1). However, it should be noted that despite the salt‐induced decrease, the transcript levels of *PcHA4* were still higher in fungus‐colonized than in NM roots under salt stress.

**Figure 5:**
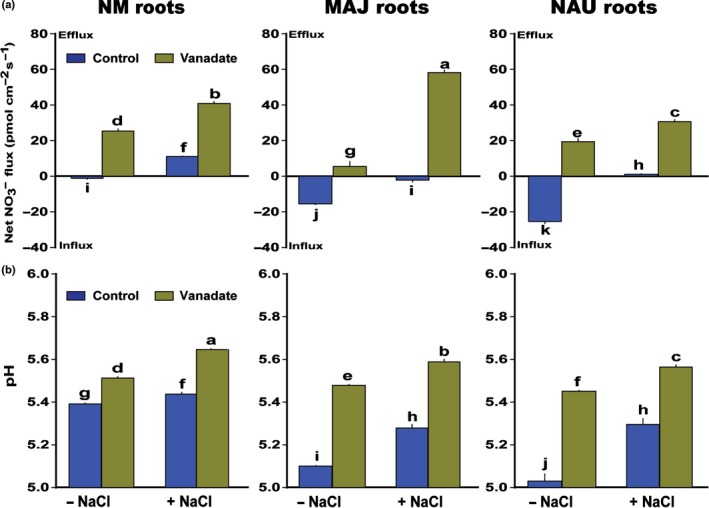
Effects of orthovanadate on steady‐state fluxes of nitrate (NO_3_^−^) and root surface pH in *Populus × canescens* in the presence and absence of *Paxillus involutus* (MAJ and NAU) and sodium chloride (NaCl). *P. × canescens* roots were inoculated without or with the *P. involutus* strains (MAJ and NAU) for 30 d, respectively. Nonmycorrhizal (NM) and fungus‐colonized *P. × canescens* plants were exposed to 0 or 100 mM NaCl for 24 h in MS nutrient solution. Before pH and flux recordings, these roots were pretreated with 500 μM sodium orthovanadate for 40 min. (a, b) Mean values of NO_3_^−^ and pH that were measured along root axis, 100–2100 μm from the apex, at intervals of 100–300 μm (Supporting Information Fig. S6). Each column is the mean of five or six individual plants, and bars represent the SEM. Columns labelled with different letters indicate significant differences at *P *<* *0.05 between treatments.

To test the importance of H^+^ gradients for NO_3_^−^ uptake, HAs were inhibited with orthovanadate. The inhibitor significantly increased the pH at the surface of NM and fungus‐colonized roots (Figs 5b, S6b), indicating that H^+^ pumps were effectively suppressed. Measurements of H^+^ fluxes confirmed that the inhibitor orthovanadate shifted net H^+^ efflux towards influx under control and saline conditions (Fig. S7). This resulted in NO_3_^−^ release regardless of the presence or absence of *Paxillus* or salt stress (Figs 5a, S6a).

In addition to pharmacological tests, membrane potential and O_2_ uptake were examined to testify whether fungal‐elicited pH changes were due to HA activity or elevated respiration. *P. involutus*‐colonized roots exhibited higher hyperpolarization of the plasma membrane (−70 to −80 mV), compared with NM roots (−50 mV; Fig. S8). The more negative membrane potential indicates H^+^‐pumping activity in the PM. It is also possible that an elevated root respiration leads to CO_2_ formation, which is reacting with water to give carbonic acid, thus resulting in acidification. O_2_ uptake rates along roots were similar in *P. involutus*‐colonized and noncolonized roots, although MAJ‐roots exhibited higher uptake values at 500–900 μm from the tip (Fig. S9). The small increase in respiration could not cause such an obvious acidification in mycorrhizal roots (Figs 1b, S3b).

## Discussion

### *Paxillus involutus* colonization enhances root NO_3_^−^ uptake in the presence or absence of a Hartig net

A main result of this study was that both *P. involutus* strains, MAJ and NAU, enhanced net NO_3_^−^‐fluxes under control conditions. This finding was further supported by a ^15^N‐NO_3_^−^ labelling experiment, where ^15^N was enriched in root and shoots of mycorrhizal plants compared with NM plants. This outcome was unexpected, because only MAJ forms a functional ectomycorrhiza, whereas the Hartig net is absent in roots colonized by NAU (poplar: this study; Gafur *et al*., 2004; birch: Le Quéré *et al*., 2004). Both MAJ and NAU colonization also resulted in upregulation of *NRT*s, which are responsible for NO_3_^−^ uptake (Wang *et al*., 2012). There were no fungal effects on NO_3_^−^‐ or nitrite‐reductase activities in roots, which might have been expected when NO_3_^−^ is stored under sufficient nutrition or transported to leaves for reduction and assimilation. Similarly, in *P. pinaster* colonized with *H. cylindrosporum*, host nitrite reductase transcript levels were only transiently induced by NO_3_^−^ (Bailly *et al*., 2007). Collectively, enhancement of NO_3_^−^ fluxes (this study; Plassard *et al*., 2002; Gobert & Plassard, 2002) and upregulation of NRTs (this study; Willmann *et al*., 2014) in EM root systems may contribute to the widely observed mycorrhizal improvement of N nutrition (Nehls & Plassard, 2018).

Our study revealed complex mechanisms underlying the fungal modification of net NO_3_^−^ fluxes. In NM and NAU‐colonized roots, net NO_3_^−^ fluxes showed moderate pH sensitivity, implying that similar uptake systems were physiologically active, but the shift of the flux/H^+^ curves towards higher net NO_3_^−^ uptake in NAU‐colonized roots may point to a higher number of active NTRs. This proposal is supported by higher gene expression of *NRT1.1* and *NRT2.1* in those roots. NRT1 and NRT2 family members are mostly PM‐located H^+^ NO_3_^−^ symporters (Glass *et al*., 1992; Espen *et al*., 2004; Xu *et al*., 2012), whose function requires an electrochemical H^+^ gradient across the membrane (Santi *et al*., 1995; Miller & Smith, 1996; Santi, 2003; Britto & Kronzucker, 2006; Sorgonà *et al*., 2010, 2011). Correspondingly, NAU‐colonized roots exhibited higher root surface acidity than NM root did, supporting facilitated NO_3_^−^ influx.

Our NO_3_^−^ flux/H^+^ curves and *NRT* transcript abundances indicated that the same factors as in NAU‐colonized roots (lower root surface pH, higher number of transporters) also drove higher net NO_3_^−^ influx in MAJ‐colonized than in NM roots. In addition, the MAJ‐colonized roots showed a higher apparent pH sensitivity of net NO_3_^−^ uptake, meaning that a small decrease in pH resulted in substantially higher changes in net NO_3_^−^ flux than in either NAU‐colonized or NM roots. We can currently only speculate about the underlying mechanisms. Poplars possess a large number of genes for potential high‐ and low‐affinity NRTs (> 70), many of them expressed in roots (Bai *et al*., 2013). For example, in *P. tremula* × *tremuloides*,* PttNRT2.5B* expression was specifically activated in symbiosis with *Amanita muscaria* (Willmann *et al*., 2014). It is possible that *Paxillus* MAJ also activated specific NRTs (not stimulated by NAU) with an increased affinity for NO_3_^−^ or with an altered H^+^/NO_3_^−^ stoichiometry, although the generally accepted ratio for the H^+^ : NO_3_^−^ symport is 2 : 1 (Meharg & Blatt, 1995). However, exceptions have been found. For instance, an increased pH, which caused a marked reduction in the maximum NO_3_^−^ current in *Arabidopsis* and in hyphae of the mold fungus *Neurospora crassa*, resulted in an increased apparent affinity for NO_3_^−^ (Meharg & Blatt, 1995; Blatt *et al*., 1997). Compared with NM roots, the higher slope in MAJ‐colonized roots could, thus, be a consequence of a more efficient charge‐coupling stoichiometry for NO_3_^−^ uptake, as demonstrated by voltage clamp and NO_3_^−^‐selective macroelectrode measurements in *N. crassa* (charge stoichiometry for NO_3_^−^ transport of 1 H^+^ : 1 NO_3_^−^, Blatt *et al*., 1997). Another possibility is that the Hartig net, which existed only in MAJ‐colonized roots, influenced NO_3_^−^ flux behavior. For example, even in the absence of salt stress, MAJ‐colonized roots contain higher sodium ion (Na^+^) concentrations than NM roots do (Langenfeld‐Heyser *et al*., 2007). Since Na^+^ leads to membrane depolarization (Sun *et al*., 2010, 2012), and here NaCl exposure also affected the apparent pH sensitivity of net NO_3_^−^ fluxes, effects on the membrane potential of MAJ‐colonized roots might have modified NO_3_^−^ uptake. However, these ideas are currently speculative and will have to be clarified in future studies.

*Paxillus involutus* can grow on NO_3_^−^ as sole N source, indicating its ability for NO_3_^−^ uptake. Here, we found an NO_3_^−^ influx into pure mycelia of MAJ and NAU, supporting that the *P. involutus* has the ability for NO_3_^−^ uptake. In previous studies, NO_3_^−^ uptake was also found in pure fungal cultures of *R. roseolus*, which had been NO_3_^−^ starved for 7 d before the measurements (Gobert & Plassard, 2002; Plassard *et al*., 2002). The form, in which N is translocated from the fungus to the plant, is still under study. Earlier studies tracing the compound‐specific fate of N supported that a large fraction of supplied ^15^N‐NO_3_^−^ was translocated by *P. involutus* without metabolization, whereas ^15^N‐ammonium ion (NH_4_^+^) was strongly assimilated by the mycorrhiza and translocated in form of amino acids or ^15^N‐NH_4_^+^ to the host (birch, pine: Ek *et al*., 1994). Similarly, in field and glasshouse studies, ectomycorrhizas colonized by mixed fungal species strongly accumulated ^15^N from labelled NH_4_^+^ but little from labelled NO_3_^−^, whereas the host trees showed the opposite behavior (Leberecht *et al*., 2016; Nguyen *et al*., 2017). Taken together, these studies advocate direct NO_3_^−^ transfer from hyphae to the host roots and stimulation of host NRT activities by *P. involutus*, but more studies are required to clarify this point.

### *Paxillus involutus* colonization ameliorates root NO_3_^−^ uptake in presence of salt stress

Under salt stress, EM trees generally exhibit improved plant growth and retain a better N nutrition status in roots and leaves compared with NM trees (Chen & Polle, 2010; Chen *et al*., 2014). NO_3_^−^ appears to play a central role for the amelioration of negative salt effects, because NO_3_^−^ (1 mM) addition to the growth medium reduced the inhibition of poplar root growth (Ehlting *et al*., 2007). Moreover, the salt‐altered N metabolism in *P*. × *canescens* was ameliorated by NO_3_^−^ supplement (Dluzniewska *et al*., 2007; Ehlting *et al*., 2007). Here, pure fungal mycelia showed NO_3_^−^ entry under salt stress similar to that of nonstressed mycelia. Therefore, we infer that fungal handling of NO_3_^−^ was not or only little affected by high salt. In agreement with other studies, salt stress caused strong net NO_3_^−^ efflux from NM roots (Yu *et al*., 2016; Dai *et al*., 2015), which could obviously not be compensated by increasing NRT gene expression. The upregulation of *PcNRT*s under salt stress might have been induced by the depletion of NO_3_^−^ in the roots, because low NO_3_^−^ availability drastically stimulates various members of the *NRT* gene families in poplar (Willmann *et al*., 2014; Gan *et al*., 2016). By contrast, with NM roots, fungal colonization largely prevented NO_3_^−^ efflux under salt stress, and in most cases a moderate influx was maintained. These findings underpin that colonization of roots with *P. involutus* constitutes an important contribution to host plant adaptation to salt‐affected environments by keeping up NO_3_^−^ nutrition.

As a result of salt exposure, the curves for the dependence of NO_3_^−^ fluxes on root surface pH of fungal‐colonized roots became more similar to those of NM roots. This finding suggests that the beneficial effects of fungal colonization that had led to shifts of NO_3_^−^ fluxes towards net uptake and elevated *PcNRT* gene expression levels under control conditions were overruled by salt stress, implying that the ameliorative effect of fungi on NO_3_^−^ flux under salt was caused by their influence on root surface pH. This conclusion was further supported by our finding that HA inhibition abolished the pH differences under salt stress and resulted in strong efflux of NO_3_^−^, regardless of fungal colonization.

### Functional significance of PM HA for NO_3_^−^ uptake

PM‐localized H^+^ pumps are essential for plant nutrient uptake (Shabala & Cuin, 2008). For NO_3_^−^ nutrition, Lupini *et al*. (2016) demonstrated temporal and spatial correlations between NO_3_^−^ influx, H^+^ efflux, and expression of PM HA genes, *MHA3* and *MHA4*, along the primary maize roots. A key result of our study was that NO_3_^−^ influx and retention under salt stress were mainly caused by the pronounced fungal‐induced activation of PM HAs in the host roots. Fungal activation of host H^+^ pumps appears to be a common component for mycorrhizal‐enhanced stress tolerance. For example, activation of PM HAs in poplar roots by *P. involutus* also played a role in maintaining potassium homeostasis under salt stress and improving cadmium tolerance (Li *et al*., 2012a; Ma *et al*., 2014b,b; Zhang *et al*., 2017). Similarly, in *Pinus sylvestris*–*Laccaria laccata* EM association, strong PM ATPase activity was observed in root cortical cells, external hyphae, sheaths, and the Hartig net (Lei & Dexheimer, 1988). For arbuscular mycorrhizal associations, some host PM HA isoforms show increased activity and gene expression after fungal colonization (Ferrol *et al*., 2002; Ramos *et al*., 2005; Rosewarne *et al*., 2007). *In situ* hybridization using full‐length probes showed higher expression of *LHA1*,* LHA2*, and *LHA4* in *Glomus intraradices*‐colonized tomato (*Lycopersicon esculentum*) roots when compared with NM plants (Rosewarne *et al*., 2007). In *Arabidopsis thaliana*, AHA4 stimulates H^+^ extrusion and enhances salt tolerance (Vitart *et al*., 2001; Gévaudant *et al*., 2007). Our data suggest that an increased expression of *PcHA4* may have led to high activities of PM HAs and enhanced surface acidification in MAJ‐colonized roots. We found that NAU also enhanced expression of *PcHA8* and *PcHA11* under salinity stress, in addition to the *PcHA4* stimulation. These results suggest that higher stimulation of host H^+^‐pumps by the strain NAU might have led to the observed higher acidification of root surfaces than that induced by the strain MAJ. However, at present, we cannot exclude the possibility that temporally increased *PcNRT1.1* and *PcHA4/HA8* transcript levels of *Paxillus* NAU‐infected roots could also have been the result of salt stress responses, due to the thin fungal mantle and, thus, limited shielding of the root.

### Conclusions

Here, we demonstrated that poplar roots colonized with the EM fungus *P. involutus* exhibited enhanced net NO_3_^−^ uptake and increased expression of several *NRTs* and *HAs*. Surprisingly, a Hartig net, which is thought to form the main exchange interface for nutrients between host and fungus, was not required for improved NO_3_^−^ flux. The *P. involutus* strain NAU (no Hartig net) caused enhanced net NO_3_^−^ fluxes and increases in *NRT* and *HA* transcript levels similar to those in roots colonized with MAJ that form typical EM structures. The main distinction between the two strains was a higher apparent pH sensitivity of MAJ‐mediated NO_3_^−^ flux than of NAU‐mediated NO_3_^−^ flux.

Beneficial effects of the fungi that we ascribed to enhanced NO_3_^−^ transport capacities were overruled by salt stress. Still, the *Paxillus* strains MAJ and NAU fostered the maintenance of root NO_3_^−^ homeostasis under salt stress due to higher surface acidity of fungus‐colonized roots than of NM roots. Salt‐stress‐induced NO_3_^−^ efflux from NM roots was combatted by mycorrhizal activated HAs, which apparently caused an H^+^ gradient across the plasma membrane sufficient for NO_3_^−^ retention. The nature of how mycorrhizal fungi can influence H^+^‐pumping activities and the transcriptional regulation of host *PcNRT*s and *PcHA*s needs to be explored in the future. Overall, the results of this study open new insights into the functioning of mycorrhizal symbioses for nutrient uptake and stress amelioration.

## Author contributions

GS, SC, and JY designed research; GS, JY, CD, JL, Yinan Zhang, ZZ, Yuhong Zhang and XM performed experiments; GS, JY, CD, JL, Yinan Zhang, RZ, SL, CL, AP and SC analyzed results; and GS, JY, AP and SC wrote the manuscript. GS, JY, CD and JL contributed equally to this work.

## Supporting information

Please note: Wiley Blackwell are not responsible for the content or functionality of any Supporting Information supplied by the authors. Any queries (other than missing material) should be directed to the *New Phytologist* Central Office.

**Fig. S1** Surface pH and steady‐state H^+^ flux at the apical region (300 or 400 μm from the tip) of *Populus* × *canescens* roots colonized without (NM) or with *Paxillus involutus* strains MAJ and NAU.
**Fig. S2** Root transverse section of *Populus × canescens* colonized without (NM) or with *Paxillus involutus* strains MAJ and NAU.
**Fig. S3** Effects of NaCl on steady‐state fluxes of NO_3_^−^ and root surface pH in *Populus × canescens* colonized without (NM) or with *Paxillus involutus* strains MAJ and NAU.
**Fig. S4** Effects of NaCl on steady NO_3_^−^ flux and surface pH in *Paxillus involutus* strains MAJ and NAU.
**Fig. S5** Effects of NaCl on steady‐state fluxes of H^+^ in *Populus *×* canescens* roots colonized without (NM) or with *Paxillus involutus* strains MAJ and NAU.
**Fig. S6** Effects of orthovanadate on steady‐state fluxes of NO_3_^−^ and root surface pH in *Populus × canescens* colonized without (NM) or with *Paxillus involutus* strains MAJ and NAU under NaCl stress.
**Fig. S7** Effects of orthovanadate on steady‐state fluxes of H^+^ in *Populus *×* canescens* colonized without (NM) or with *Paxillus involutus* strains MAJ and NAU under NaCl stress.
**Fig. S8** Membrane potential of *Populus *×* canescens* roots colonized without (NM) or with *Paxillus involutus* strains MAJ and NAU.
**Fig. S9** Oxygen flux in *Populus *×* canescens* roots colonized without (NM) or with *Paxillus involutus* strains MAJ and NAU.
**Table S1** Nernst slope and intercept of the H^+^ microelectrodes in H^+^ and NO_3_^−^ measuring solutions.
**Table S2** Primer sets used for quantitative real‐time PCR.
