# Supplementary material for: Amelioration of nitrate uptake under salt stress by ectomycorrhiza with and without a Hartig net
Source: New Phytol. 2019 Mar 14;222(4):1951–64. doi: 10.1111/nph.15740 (PMC6594093; doi:10.1111/nph.15740)
Supplement: Supplementary file 1 — Fig. S1 Surface pH and steady‐state H+ flux at the apical region (300 or 400 μm from the tip) of Populus × canescens roots colonized without (NM) or with Paxillus involutus strains MAJ and NAU. Fig. S2 Root transverse section of Populus × canescens colonized without (NM) or with Paxillus involutus strains MAJ and NAU. Fig. S3 Effects of NaCl on steady‐state fluxes of NO3 − and root surface pH in Populus × canescens colonized without (NM) or with Paxillus involutus strains MAJ and NAU. Fig. S4 Effects of NaCl on steady NO3 − flux and surface pH in Paxillus involutus strains MAJ and NAU. Fig. S5 Effects of NaCl on steady‐state fluxes of H+ in Populus × canescens roots colonized without (NM) or with Paxillus involutus strains MAJ and NAU. Fig. S6 Effects of orthovanadate on steady‐state fluxes of NO3 − and root surface pH in Populus × canescens colonized without (NM) or with Paxillus involutus strains MAJ and NAU under NaCl stress. Fig. S7 Effects of orthovanadate on steady‐state fluxes of H+ in Populus × canescens colonized without (NM) or with Paxillus involutus strains MAJ and NAU under NaCl stress. Fig. S8 Membrane potential of Populus × canescens roots colonized without (NM) or with Paxillus involutus strains MAJ and NAU. Fig. S9 Oxygen flux in Populus × canescens roots colonized without (NM) or with Paxillus involutus strains MAJ and NAU. Table S1 Nernst slope and intercept of the H+ microelectrodes in H+ and NO3 − measuring solutions. Table S2 Primer sets used for quantitative real‐time PCR. [file NPH-222-1951-s001.pdf]

New Phytologist

***New Phytologist* Supporting Information**

Article title: Amelioration of nitrate uptake under salt stress by ectomycorrhiza with and without a Hartig net

Authors: Gang Sa, Jun Yao, Chen Deng, Jian Liu, Yinan Zhang, Zhimei Zhu, Yuhong Zhang, Xujun Ma, Rui Zhao, Shanzhi Lin, Cunfu Lu, Andrea Polle, Shaoliang Chen

Article acceptance date: 01 February 2019

The following Supporting Information is available for this article:

Fig. S1 Surface pH and steady-state H^+ flux at the apical region (300 or 400 µm from the tip) of *Populus* × *canescens* roots colonized without (NM) or with *Paxillus involutus* strains MAJ and NAU.

Fig. S2 Root transverse section of *Populus* × *canescens* colonized without (NM) or with *Paxillus involutus* strains MAJ and NAU.

Fig. S3 Effects of NaCl on steady-state fluxes of NO_3^- and root surface pH in *Populus* × *canescens* colonized without (NM) or with *Paxillus involutus* strains MAJ and NAU.

Fig. S4 Effects of NaCl on steady NO_3^- flux and pH in *Paxillus involutus* strains MAJ and NAU.

Fig. S5 Effects of NaCl on steady-state fluxes of H^+ in *Populus* × *canescens* roots colonized without (NM) or with *Paxillus involutus* strains MAJ and NAU.

Fig. S6 Effects of orthovanadate on steady-state fluxes of NO_3^- and root surface pH in *Populus* × *canescens* colonized without (NM) or with *Paxillus involutus* strains MAJ and NAU under NaCl stress.

Fig. S7 Effects of orthovanadate on steady-state fluxes of H^+ in *Populus* × *canescens* colonized without (NM) or with *Paxillus involutus* strains MAJ and NAU under NaCl stress.

Fig. S8 Membrane potential of *Populus* × *canescens* roots colonized without (NM) or with *Paxillus involutus* strains MAJ and NAU.

Fig. S9 Oxygen flux in *Populus* × *canescens* roots colonized without (NM) or with *Paxillus involutus* strains MAJ and NAU.

Table S1 Nernst slope and intercept of the H^+ microelectrodes in H^+ and NO_3^- measuring solutions.

Table S2 Primer sets used for quantitative real-time PCR.

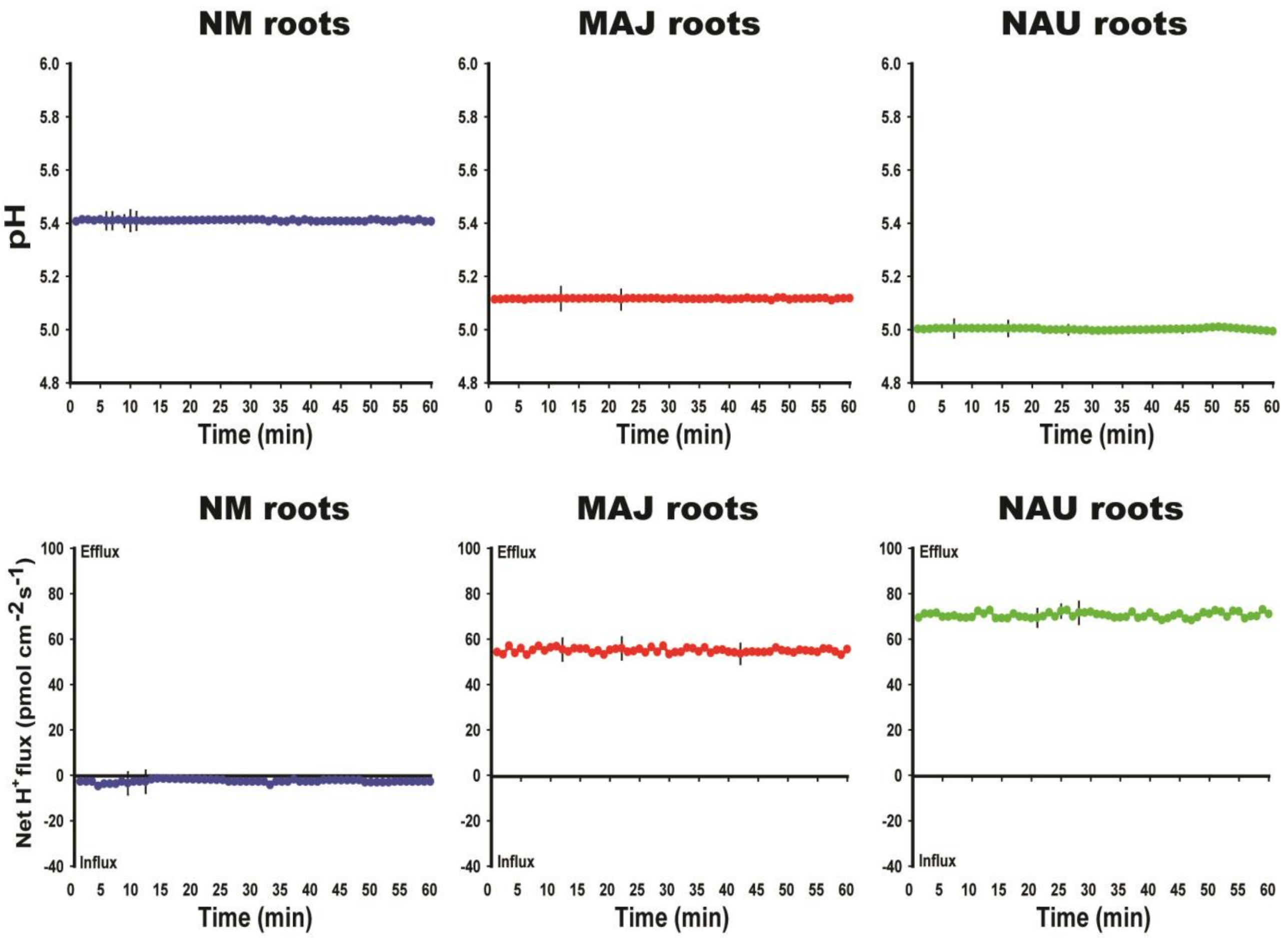

Fig. S1 Surface pH and steady-state H^+ flux at the apical region (300 or 400 µm from the tip) of *Populus* × *canescens* roots colonized without (NM) or with *Paxillus involutus* strains MAJ and NAU. Each point is the mean of five to six individual plants, and bars represent the standard error of the mean (When error bars are not visible, they were smaller than the symbols).

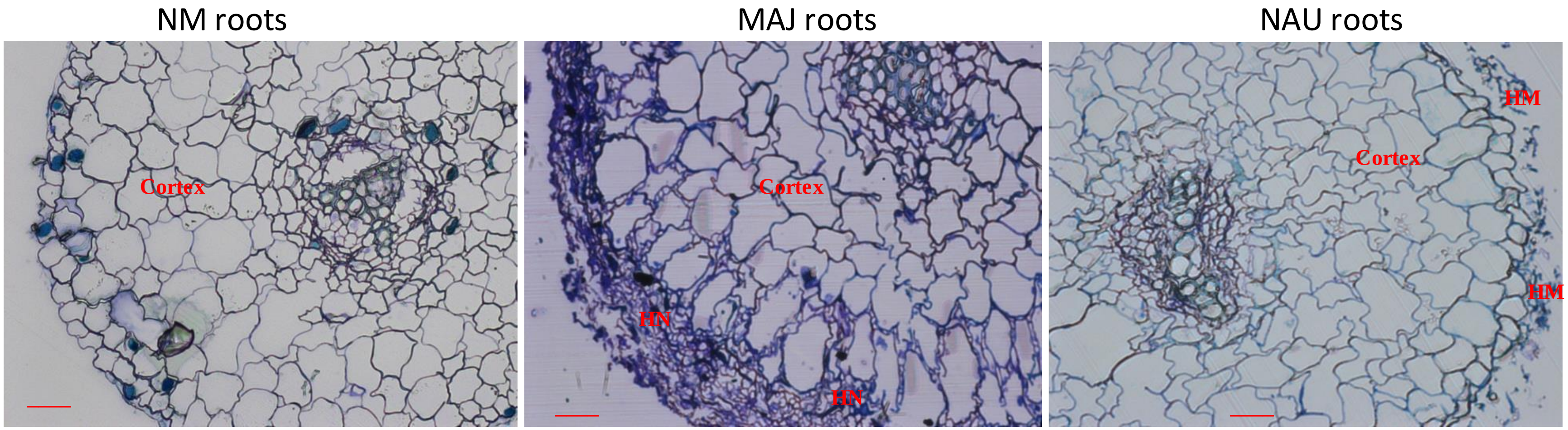

Fig. S2 Root transverse section of *Populus* × *canescens* colonized without (NM) or with *Paxillus involutus* strains MAJ and NAU. Semi-thin sections (500 nm) of the tissues were stained with 0.1% (w/v) toluidine blue in 0.1% (w/v) disodium tetraborate decahydrate for light microscopy (Axioplan, Carl Zeiss, Oberkochen, Germany). Microphotographs were taken with a digital camera (Coolpix 990, Nikon, Tokyo). HN = hyphal net or Hartig net, HM = hyphal mantel. Scale bar = 20 µm.

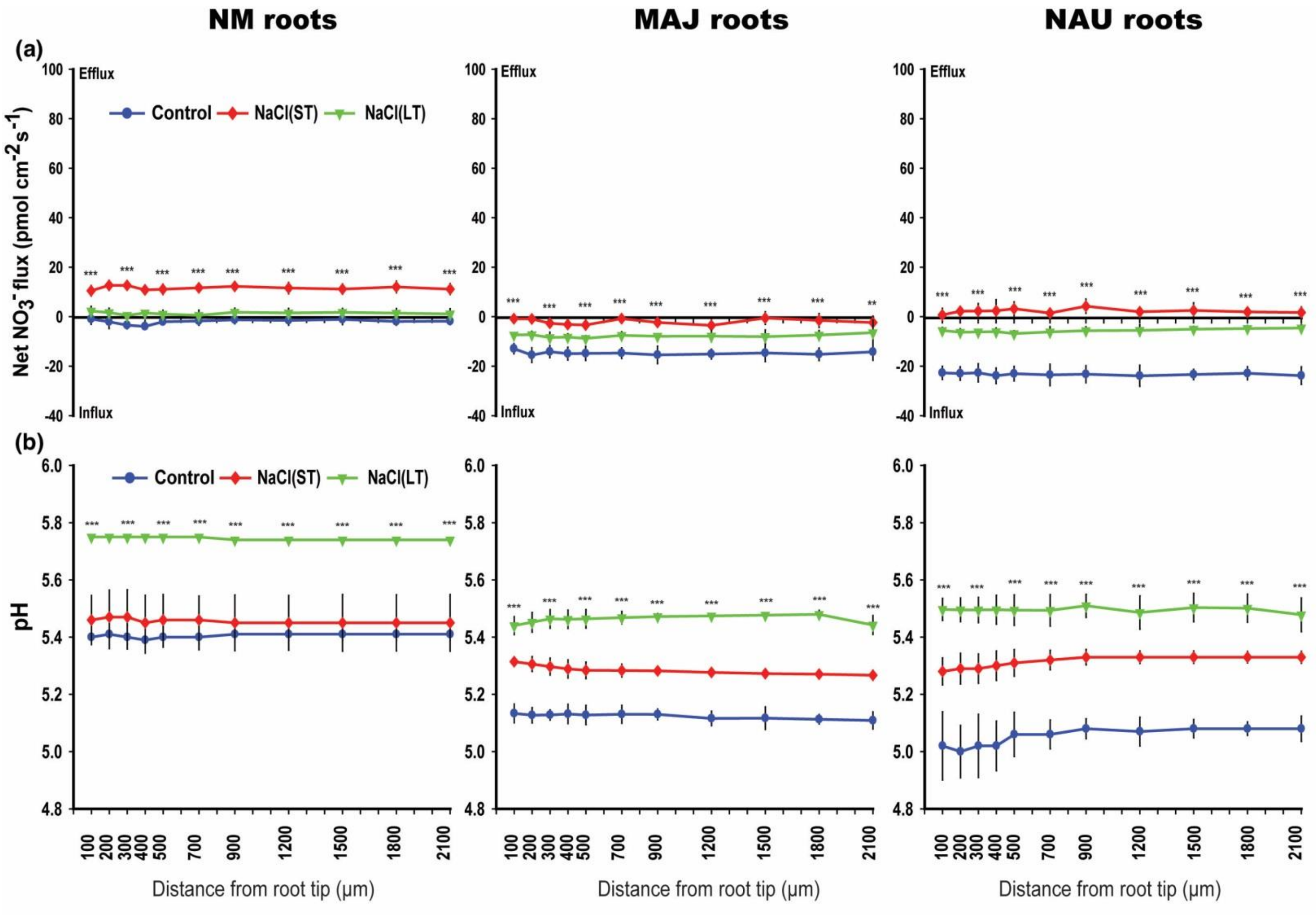

Fig. S3 Effects of NaCl on steady-state fluxes of NO_3^- and root surface pH in *Populus* × *canescens* colonized without (NM) or with *Paxillus involutus* strains MAJ and NAU. (a) NO_3^- fluxes; (b) Root surface pH. *P.* × *canescens* roots were inoculated without or with the *P. involutus* strains (MAJ and NAU) for 30 days, respectively. NM and fungus-colonized *P.* × *canescens* plants were exposed to 0 or 100 mM NaCl for 24 h (short-term, ST) or 7 d (long-term, LT) in MS nutrient solution. NO_3^- fluxes and pH values were measured along root axis, 100–2,100 µm from the apex, at intervals of 100 to 300 µm. Each point is the mean of five to six individual plants, and bars represent the standard error of the mean. $^{**}P < 0.01$, $^{***}P < 0.001$ compared to no-salt controls.

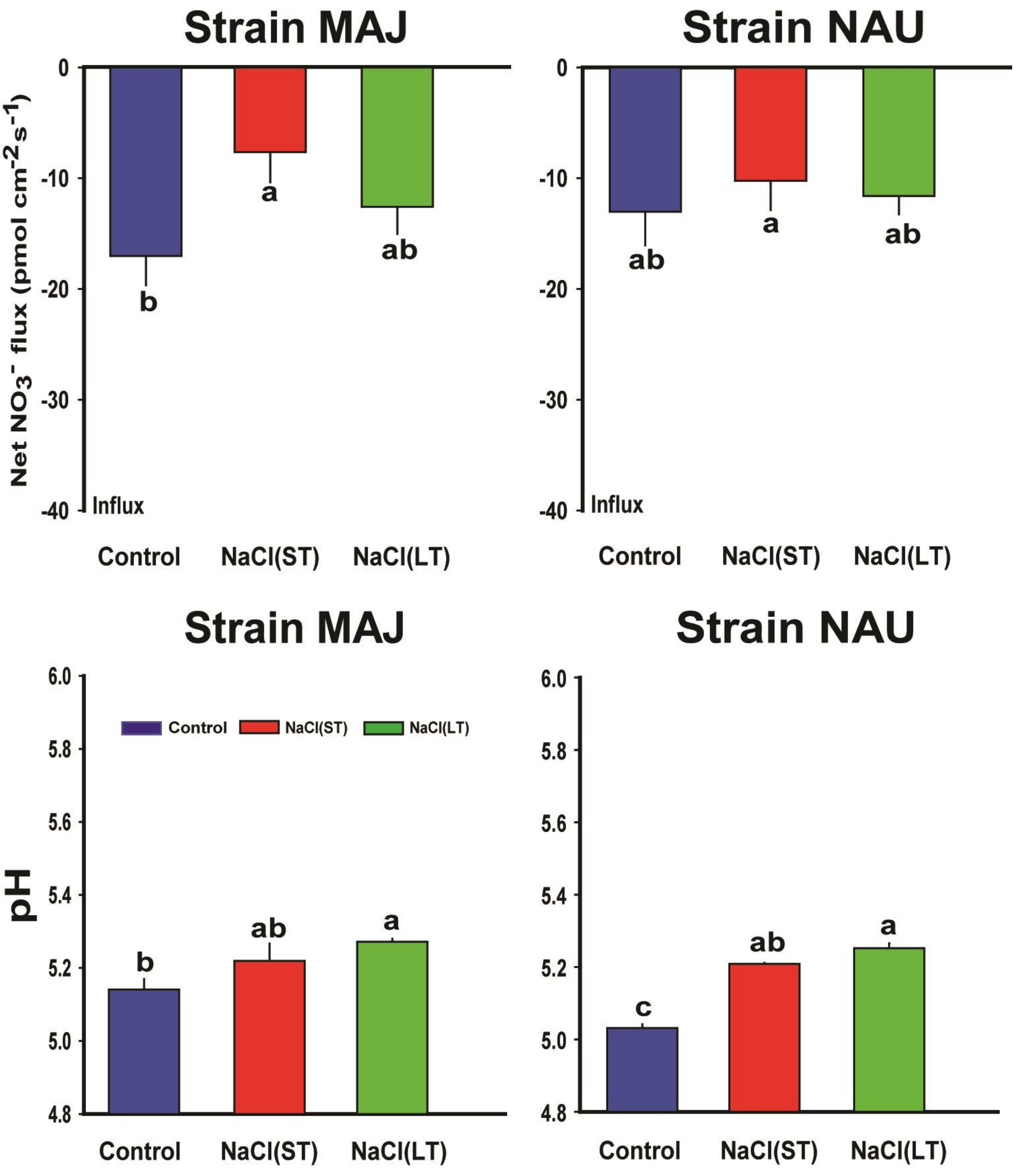

Fig. S4 Effects of NaCl on steady NO_3^- flux and surface pH in *Paxillus involutus* strains MAJ and NAU. MAJ and NAU mycelia (the youngest and active hyphae) were subjected to 0 or 100 mM NaCl for 24 h (short-term, ST) or 7 d (long-term, LT) in modified MS nutrient solution. Agar plugs covered by hyphae were transferred into 150 mL of measuring solutions and incubated on a rotary shaker (150 rpm, 23°C) for 1 h in darkness. Following 40 min equilibration in H^+ or NO_3^- measuring solution, NO_3^- flux and pH of mycelia were measured along the surface of hyphae over a recording period of 30 min. Each column is the mean of five to six fungal cultures, and bars represent the standard error of the mean. Columns labelled with different letters indicate significant differences at $P < 0.05$ between treatments.

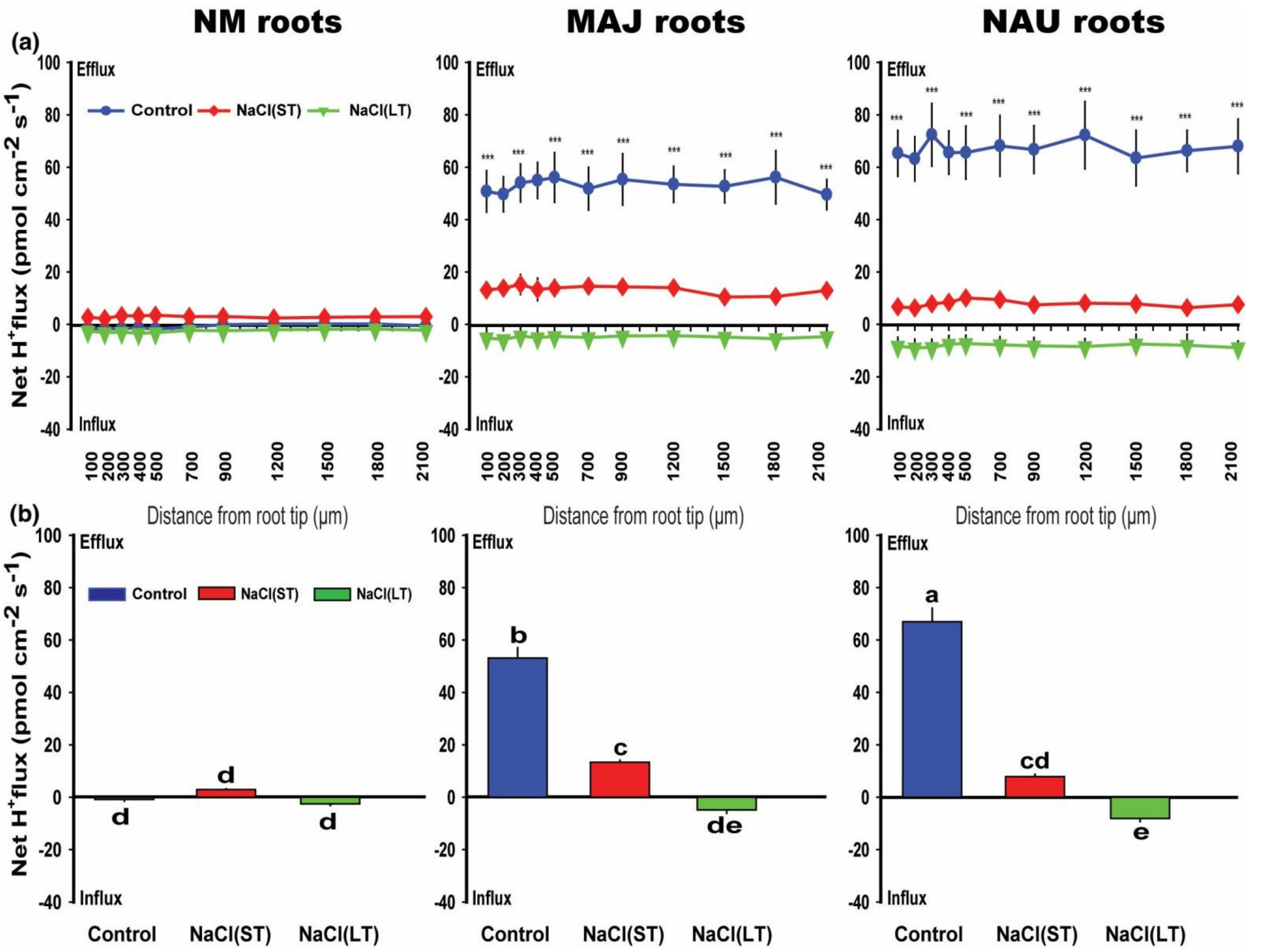

Fig. S5 Effects of NaCl on steady-state fluxes of H^+ in *Populus* × *canescens* roots colonized without (NM) or with *Paxillus involutus* strains MAJ and NAU. (a) H^+ fluxes along roots. *P.* × *canescens* roots were inoculated without or with the *P. involutus* strains (MAJ and NAU) for 30 days, respectively. NM and fungus-colonized *P.* × *canescens* plants were exposed to 0 or 100 mM NaCl for 24 h (short-term, ST) or 7 d (long-term, LT) in MS nutrient solution. H^+ fluxes were measured along root axis, 100–2,100 μm from the apex, at intervals of 100 to 300 μm. Each point is the mean of five to six individual plants, and bars represent the standard error of the mean. *** $P < 0.001$ compared to no-salt controls. (b) Mean flux rates of H^+. Columns labelled with different letters indicate significant differences at $P < 0.05$ between treatments.

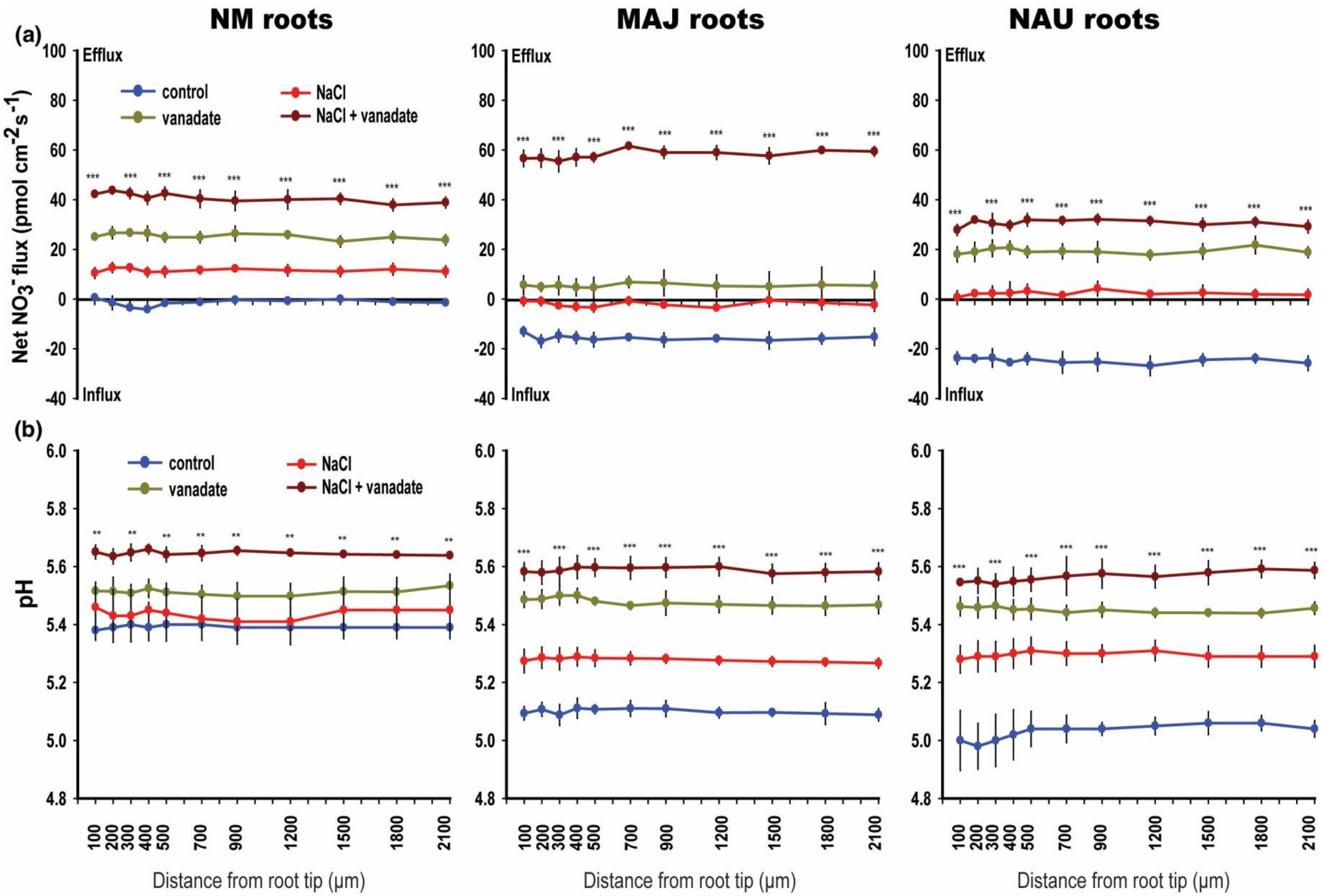

Fig. S6 Effects of orthovanadate on steady-state fluxes of NO_3^- and root surface pH in *Populus* × *canescens* colonized without (NM) or with *Paxillus involutus* strains MAJ and NAU under NaCl stress. (a) NO_3^- fluxes; (b) Root surface pH. *P.* × *canescens* roots were inoculated without or with the *P. involutus* strains (MAJ and NAU) for 30 days, respectively. NM and fungus-colonized *P.* × *canescens* plants were exposed to 0 or 100 mM NaCl for 24 h in MS nutrient solution. Prior to flux recordings, no-salt and salinized roots were pre-treated with 500 µM sodium orthovanadate for 40 min. NO_3^- fluxes and pH were measured along root axis, 100–2,100 µm from the apex, at intervals of 100 to 300 µm. Each point is the mean of five to six individual plants, and bars represent the standard error of the mean. ***P* < 0.01, ****P* < 0.001.

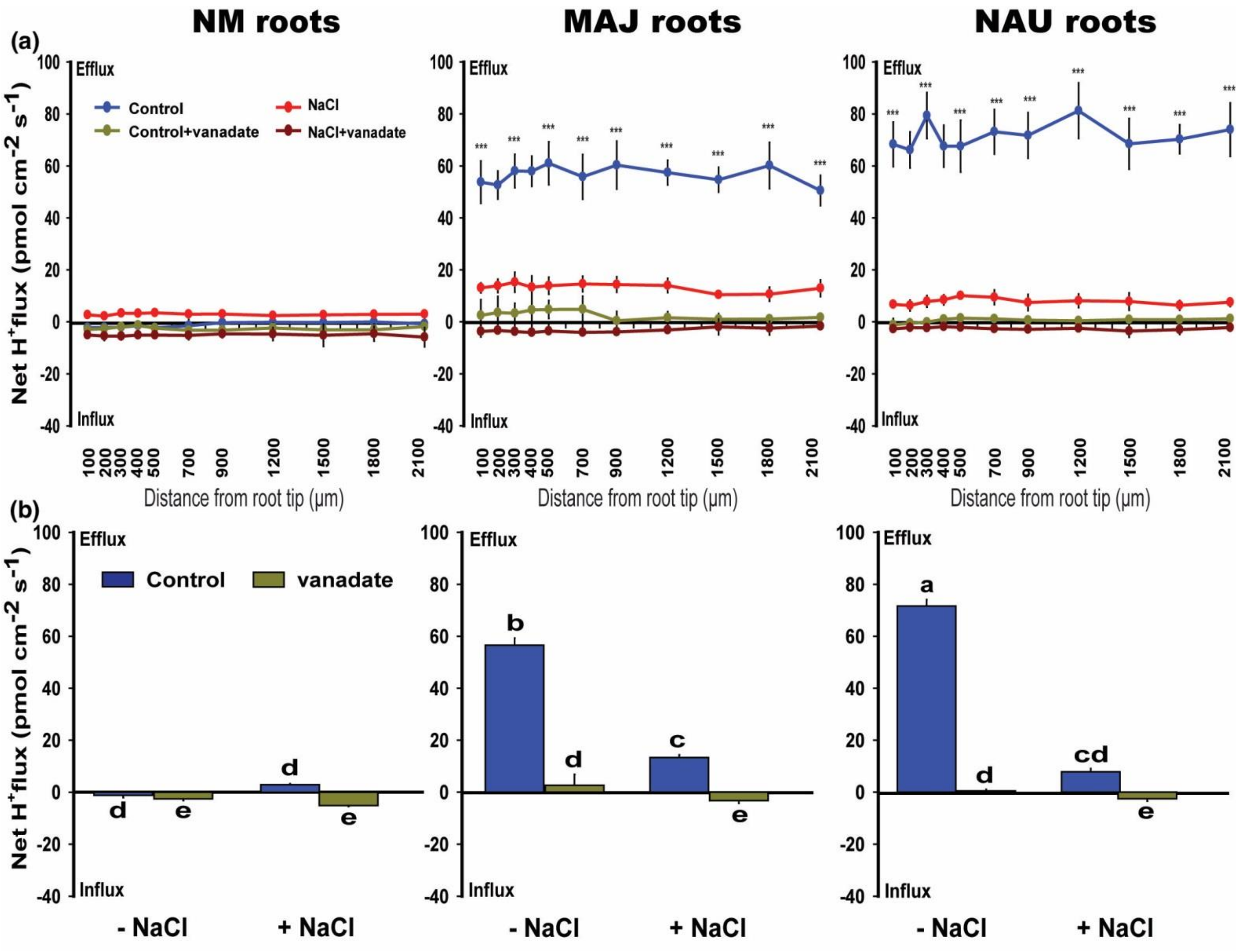

Fig. S7 Effects of orthovanadate on steady-state fluxes of H^+ flux in *Populus × canescens* colonized without (NM) or with *Paxillus involutus* strains MAJ and NAU under NaCl stress. (a) H^+ fluxes along roots. *P. × canescens* roots were inoculated without or with the *P. involutus* strains (MAJ and NAU) for 30 days, respectively. NM and fungus-colonized *P. × canescens* plants were exposed to 0 or 100 mM NaCl for 24 h in MS nutrient solution. Prior to flux recordings, no-salt and salinized roots were pre-treated with 500 μM sodium orthovanadate for 40 min. H^+ fluxes were measured along root axis, 100–2,100 μm from the apex, at intervals of 100 to 300 μm. Each point is the mean of five to six individual plants, and bars represent the standard error of the mean. *** $P < 0.001$. (b) Mean flux rates of H^+. Columns labelled with different letters indicate significant differences at $P < 0.05$ between treatments.

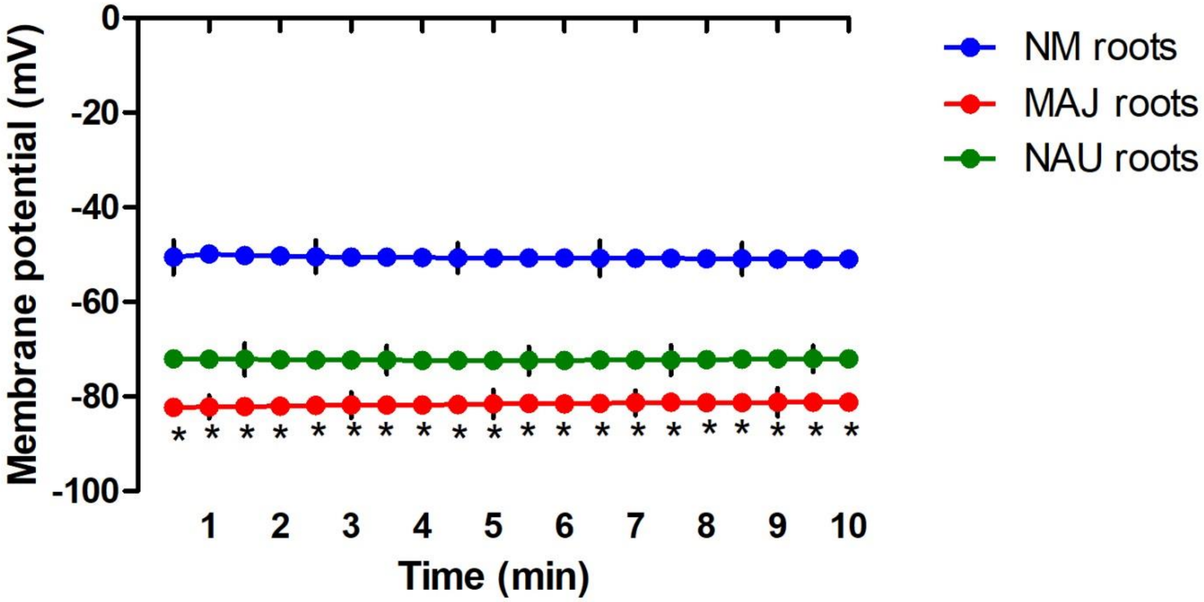

Fig. S8 Membrane potential of *Populus* × *canescens* roots colonized without (NM) or with *Paxillus involutus* strains MAJ and NAU. *P.* × *canescens* roots were inoculated without or with the *P. involutus* strains, MAJ and NAU, for 30 days, respectively. NM and *Paxillus*-colonized plants were exposed for 24 h in modified MS nutrient solution. Membrane potential of NM and mycorrhizal roots was continuously recorded for 10 min at the apical region (300 or 400 µm from the tip). Each point is the mean of five to six individual plants, and error bars represent SE.* $P < 0.05$ compared to NM roots.

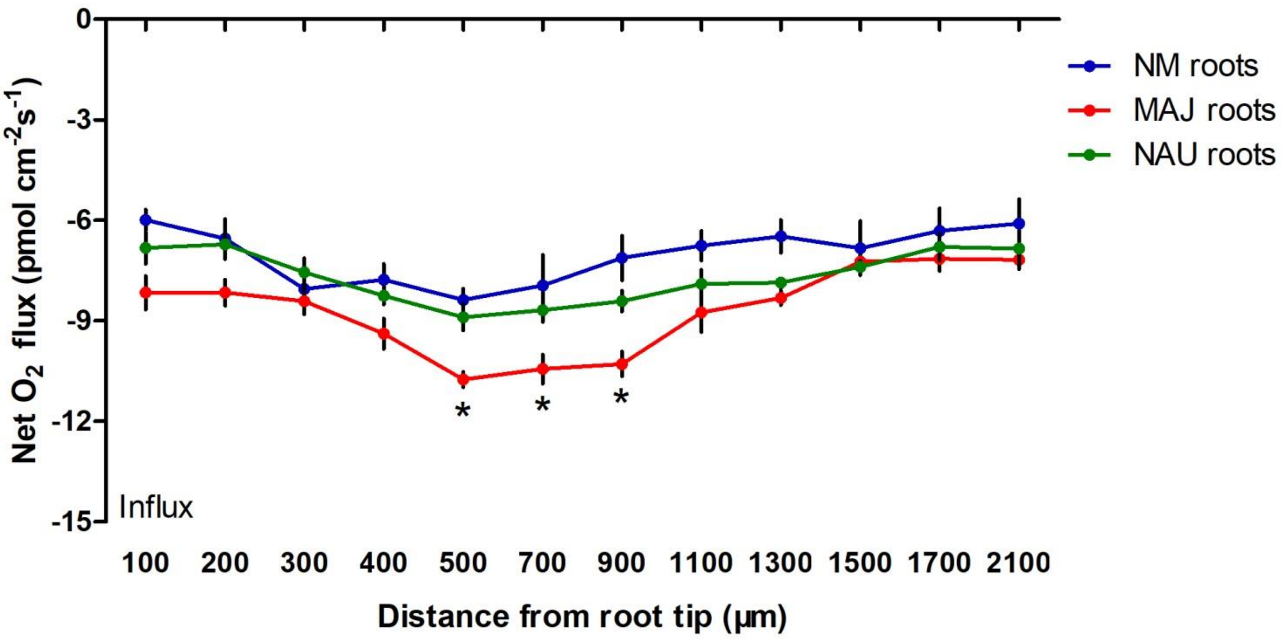

Fig. S9 Oxygen flux in *Populus* × *canescens* roots colonized without (NM) or with *Paxillus involutus* strains MAJ and NAU. *P.* × *canescens* roots were inoculated without or with the *P. involutus* strains, MAJ and NAU, for 30 days, respectively. NM and *Paxillus*-colonized plants were exposed for 24 h in modified MS nutrient solution. Flux recordings of O_2 started at a distance of 100 μm from the apex and were conducted along the root axis until 2,100 μm from the apex at intervals of 100 to 400 μm. Each point is the mean of five to six individual plants, and error bars represent SE.* $P < 0.05$ compared to NM roots.

Table S1 Nernst slope and intercept of the H^+ microelectrodes in H^+ and NO_3^- measuring solutions. H^+-selective microelectrodes were calibrated in H^+ measuring solution (0.1 mM NaCl, 0.1 mM $MgCl_2$, 0.1 mM $CaCl_2$ and 0.5 mM KCl) and NO_3^- measuring solution (0.1 mM KNO_3, 0.1 mM KCl, 0.1 mM $CaCl_2$) at pH 4.5, 5.5, and 6.5, respectively. Each value is the mean of five to six measurements, and error values represent ±SE.

	H^+ measuring solution	NO_3^- measuring solution
Nernst Slope	58.6±1.8	59.0±2.0
Nernst Intercept	364.5±3.1	366.0±2.9

Table S2 Primer sets used for quantitative real-time PCR

Gene Name	GeneBank Accession NO. (*Populus trichocarpa*)	Arabidopsis Homolog locus	Primer sets	Sequence (5' to 3')
PcUBQ-L	XM_002307155	AT1G31340	Forward Primer	TGAGGCTTAGGGGAGGAACT
			Reverse Primer	TGTAGTCGCGAGCTGTCTTG
PcNRT1.1	XM_002303476	AT1G12110	Forward Primer	AGGTGGAGAGGCAATGGAGAG
			Reverse Primer	GTGGCGAAGATGGCGATGG
PcNRT1.2	XM_024609537	AT1G69850	Forward Primer	GTGGCGAAGATGGCGATGG
			Reverse Primer	AAGCACAGAACTGAGGTAGTATCC
PcNRT2.1	XM_002313493	AT1G08090	Forward Primer	TGTGGTACTTGGTGGTGT
			Reverse Primer	TGTGGTACTTGGTGGTGT
PcHA4	XM_006382102	AT3G47950	Forward Primer	TGTGGTACTTGGTGGTGT
			Reverse Primer	CCTAAATGACGGAACAAT
PcHA8	XM_024582562	AT3G42640	Forward Primer	AGCAAGAATCCCAGCACA
			Reverse Primer	AGCAAGAATCCCAGCACA
PcHA11	XM_024582562	AT5G62670	Forward Primer	CTGATTGAGTTCGGTGAA
			Reverse Primer	CTGATTGAGTTCGGTGAA
